# Clinical features, investigations, and outcomes of pediatric limbic encephalitis: A multicenter study

**DOI:** 10.1002/acn3.51494

**Published:** 2022-01-11

**Authors:** Saraswathy Sabanathan, Omar Abdel‐Mannan, Kshitij Mankad, Ata Siddiqui, Krishna Das, Lucinda Carr, Christin Eltze, Michael Eyre, Jon Gadian, Cheryl Hemingway, Marios Kaliakatsos, Rachel Kneen, Deepa Krishnakumar, Bryan Lynch, Amitav Parida, Thomas Rossor, Micheal Taylor, Evangeline Wassmer, Sukhvir Wright, Ming Lim, Yael Hacohen

**Affiliations:** ^1^ Children's Neurosciences, Evelina London Children's Hospital Guy's and St Thomas' NHS Foundation Trust London United Kingdom; ^2^ Queen Square MS Centre, UCL Queen Square Institute of Neurology, Faculty of Brain Sciences University College London London United Kingdom; ^3^ Department of Neurology Great Ormond Street Hospital for Children London United Kingdom; ^4^ Department of Neuroradiology Great Ormond Street Hospital for Children London; ^5^ Department of Neuroradiology, Evelina London Children's Hospital Guy's and St Thomas' NHS Foundation Trust London United Kingdom; ^6^ Department of Neurophysiology Great Ormond Street Hospital for Children London United Kingdom; ^7^ School of Biomedical Engineering & Imaging Sciences King's College London London United Kingdom; ^8^ Department of Paediatric Neurology King’s College Hospital NHS Foundation Trust London United Kingdom; ^9^ Department of Neurology Alder Hey Children’s NHS Foundation Trust Liverpool United Kingdom; ^10^ Department of Paediatric Neurology Addenbrooke’s Hospital Cambridge United Kingdom; ^11^ Department of Paediatric Neurology Children’s University Hospital Dublin Ireland; ^12^ Department of Neurology Birmingham Children’s Hospital Birmingham United Kingdom; ^13^ Department of Paediatric Neurology Leeds Children’s Hospital Leeds United Kingdom; ^14^ Aston Neuroscience Institute, College of Health and Life Sciences Aston University Birmingham United Kingdom; ^15^ King’s Health Partners Academic Health Science Centre London United Kingdom

## Abstract

**Objectives:**

To describe the clinical presentation, investigations, management, and disease course in pediatric autoimmune limbic encephalitis (LE).

**Methods:**

In this retrospective observational study, from the UK Childhood Neuroinflammatory Disease network, we identified children from six tertiary centers with LE <18 years old between 2008 and 2021. Clinical and paraclinical data were retrieved from medical records.

**Results:**

Twenty‐five children fulfilling LE criteria were identified, with median age of 11 years (IQR 8, 14) and median follow‐up of 24 months (IQR 18, 48). All children presented with seizures; 15/25 (60%) were admitted to intensive care. Neuroimaging demonstrated asymmetric mesial temporal changes in 8/25 (32%), and extra‐limbic changes with claustrum involvement in 9/25 (38%). None were positive for LGI1/CASPR2 antibodies (Abs), 2/25 were positive for serum anti‐NMDAR Abs, and 2/15 positive for anti‐Hu Abs; one died from relapsing neuroblastoma. Two children had serum and CSF anti‐GAD antibodies. Initial immune therapy included steroids in 23/25 (92%), intravenous immunoglobulin (IVIg) in 14/25 (56%), and plasma exchange in 7/25 (28%). The commonest second‐line treatment was rituximab in 15/25 (60%). Median duration of hospital admission was 21 days (IQR 11, 30). At last follow‐up, 13/25 (52%) had refractory seizures and 16/25 (64%) had memory impairment. Six children (24%) had modified Rankin Scale (mRS) scores ≥3. There was no significant difference in mRS, or long‐term cognitive and epilepsy outcomes in those who received rituximab versus those who did not.

**Interpretation:**

A diagnosis of autoimmune LE was associated with significant morbidity and adverse outcomes in this pediatric cohort.

## Introduction

Autoimmune limbic encephalitis (LE) is a rare but well‐recognized neuroinflammatory condition in which patients typically present with memory deficits, seizures, and psychiatric disturbances alongside radiologic changes in the medial temporal lobe.^1^, ^2^ The mean incidence rates of antibody‐mediated autoimmune encephalitis in the Netherlands between 2015 and 2018 was 1.54 children/million (95% CI 0.95–2.35).^3^ Historically, LE was first described as a paraneoplastic syndrome associated with small cell lung cancer^4^ in adult patients; however, with the discovery of antineuronal antibodies, we now know that it most frequently occurs in association with antibodies against the neuronal secreted protein leucine‐rich glioma‐inactivated (LGI1) and contactin‐associated protein‐like 2 (CASPR2).^5^, ^6^ In children, LE rarely occurs secondary to LGI1/CASPR2 antibodies, and the majority of pediatric cases are antibody negative.^3^, ^7^ This raises the question as to whether the clinical syndrome is comparable to the adult phenotype.

Prior to current understanding of antibodies in LE, a retrospective multicenter study of 10 children with LE included antibodies that are no longer considered pathogenic.^8^ The majority of studies in the pediatric literature have focused on studying autoimmune encephalitis with known antibodies against cell surface (e.g., GABA(B)R‐ab, AMPA, and glycine receptors) or synaptic proteins (e.g., GAD, LGI1 and CASPR2); an example is anti‐*N*‐methyl‐d‐aspartate receptor (anti‐NMDAR) encephalitis, the most frequently described pediatric autoimmune encephalitis (AE), in which the antibodies target the NR1 subunit of the receptor.^9^

Outcomes in pediatric AE improve with prompt recognition and treatment.^10^ Poor prognosis is associated with the need for pediatric intensive care unit (PICU) admission and status epilepticus in anti‐NMDAR encephalitis.^11^ Complete recovery in cell‐surface antibody‐mediated AE is approximately 50% in adult and pediatric cases,^10^, ^12^ and may be lower in the antibody negative group.^13^

Little is known about the natural history of autoimmune LE, particularly in the pediatric population, with only single case reports and small case series published.^8^, ^14^ Here, in a retrospective observational study, we describe the common presenting symptoms, serum and radiologic biomarkers, treatment, disease course and outcomes in 25 children with LE in the United Kingdom and the Republic of Ireland.

## Methods

The British Paediatric Neurology Association (BPNA) hosts the UK Childhood Neuro‐Inflammatory Disorders (UK‐CIND). Twenty‐three pediatric neuroscience centers participate in the UK‐CIND which keeps a database of cases discussed. LE cases were identified through the database and through direct correspondence with the network participants. Case imaging were reviewed by the two neuroradiologists (K.M. and A.S.) along with clinical information by study clinicians (O.A.‐M., Y.H., M.L., A.P., S.S., E.W., and S.W.). Diagnosis of LE was made and retrospectively confirmed again by the study team based on the following criteria^1^: (1) subacute onset (rapid progression of less than 3 months) of working memory deficits, seizures, or psychiatric symptoms suggesting involvement of the limbic system, (2) bilateral brain abnormalities on T2‐weighted fluid‐attenuated inversion recovery (FLAIR) MRI sequences highly restricted to the medial temporal lobes, (3) at least one of the following: CSF pleocytosis (white blood cell count of more than 5 cells per mm^3^) or EEG with epileptic or slow‐wave activity involving the temporal lobes, and (4) exclusion of alternative causes (infectious encephalitis, demyelinating conditions, and neurometabolic and systemic inflammatory conditions). All cases were carefully evaluated for neurotropic viruses and had negative CSF PCR for HSV and enteroviruses. Additional CSF testing for neurotropic viral DNA or RNA often performed as a panel would include varicella‐zoster virus, Epstein–Barr virus, and HHV‐6. Cases included had a minimum of 6‐month follow‐up data.

Six tertiary pediatric neurology centers contributed cases: Great Ormond Street Hospital (London), Evelina London Children's Hospital (London), Birmingham Women's and Children's Hospital, Addenbrooke's Hospital (Cambridge), Alder Hey Children's Hospital (Liverpool), and Children's University Hospital (Dublin).

Clinical data including demographics, clinical findings and laboratory results, imaging and neurophysiology reports, and treatment information were retrospectively reviewed from medical records of patients and entered in a standardized case report form. Clinical notes reported deterioration in processing information separately from difficulties retaining information and was recorded as additional cognitive difficulties. Outcomes were assessed using the modified Rankin Scale (mRS) which emphasizes gross motor skills and disability impact on age‐expected activities.^15^ This was scored on the last follow‐up data (minimum 6 months). Children with refractory seizures alone with no gross motor change or impact on age‐appropriate independence were given mRS 2. Physical motor impairment and help with activities of daily living were scored mRS 3 or more. Children admitted to PICU were scored mRS 5. Educational impact was determined by the need to change school setting to receive extra support. Neurocognitive testing was performed with standard tests of cognition dictated by clinical need. Impairment was significant if any test within these domains fell below the 5th percentile.

### Statistical analysis

Parametric or nonparametric statistical tests (Mann–Whitney *U* and Kruskal Wallis tests) were used for continuous distributions, as appropriate, and χ^2^ or Fisher's exact test for nominal data to compare the demographic characteristics, presenting symptoms, and radiologic features across the age groups. Univariate logistic regression analysis was used to predict risk factors of; (1) mRS scores of 3 or more, (2) refractory seizures, and (3) cognitive impairment at last follow up. Predictors tested included age, sex, ethnicity, intensive care admission, epileptiform discharges on EEG, abnormal CSF at presentation (defined as raised white cell count and/or protein), rituximab therapy, time from initial presentation to steroid treatment and time from initial presentation to escalation of immune therapy (IVIG/PLEX). All results associated with a *p* < 0.05 were considered significant. Data were analyzed using GraphPad Prism 9 (San Diego, CA, USA).

This study was approved by Great Ormond Street Hospital Research and Development Department (reference: 16NC10). Any data not published within the article will be shared upon request from any senior (tenured) investigator.

## Results

A total of 25 patients were identified. Four patients have been reported previously elsewhere.^10^, ^16^, ^17^ Median age at symptom onset was 11 years (range 4–15 years, IQR 8, 14 years). Fourteen patients (56%) were female. Clinical features and patient demographics are summarized in Tables 1 and 2.

**Table 1:** Demographics, baseline, and follow‐up clinical and radiologic features stratified to patients under the age of 12 years (*n* = 15) and 12–18 years (*n* = 10).

	All patients (*N* = 25)	Patients <12 years old (*n* = 15)	Patients 12–18 years old (*n* = 10)	*p* value
Male	11/25 (44%)	8/15 (53%)	3/10 (30%)	0.41
Ethnicity				
White British/Irish	8/25 (32%)	3/15 (20%)	5/10 (50%)	0.19
White other/mixed	3/25 (12%)	2/15 (13%)	1/10 (10%)	1.00
Bangladeshi/Indian/Asian	6/25 (24%)	4/15 (27%)	2/10 (20%)	1.00
Black	5/25 (20%)	3/15 (20%)	2/10 (20%)	1.00
Other	2/25 (8%)	2/15 (13%)	0/10	0.5
Not reported	1/25 (4%)	1/15 (7%)	0/10	
Median age (IQR), years	11 (8–14)	9 (7–11)	14 (13–15)	
Prodromal symptoms (antecedent 2 weeks)^1^	20/25 (80%)	14/15 (93%)	6/10 (60%)	0.12
Subacute >2 weeks but <3 months	4/25 (16%)	1/15 (6%)	3/10 (30%)	0.27
Presenting symptoms				
Fever	13/25 (52%)	11/15 (73%)	2/10 (20%)	0.02
Headache	10/25 (40%)	5/15 (33%)	5/10 (50%)	0.44
Encephalopathy	13/25 (52%)	9/15 (60%)	4/10 (40%)	0.43
Vomiting	4/25 (16%)	3/15 (20%)	1/10 (10%)	0.63
Behavior change	19/25 (76%)	13/15 (87%)	6/10 (60%)	0.18
Visual hallucinations	6/25 (24%)	3/15 (20%)	3/10 (30%)	0.65
Auditory hallucinations	5/25 (20%)	2/15 (13%)	3/10 (30%)	0.34
Short term memory impairment	20/25 (80%)	11/15 (73%)	9/10 (90%)	0.61
Additional cognitive difficulties^2^	16/25 (64%)	11/15 (73%)	5/10 (56%)	0.40
Seizures	25/25 (100%)	15/15 (100%)	10/10 (100%)	
PICU admission	16/25 (64%)	9/15 (60%)	7/10 (70%)	0.69
Seizure management	15/16 (94%)	8/9 (89%)	5/7 (71%)	0.55
Reduced consciousness	2/16 (13%)	1/9 (11%)	1/7 (14%)	1.00
To administer treatment	1/16 (6%)	0/9	1/7 (14%)	0.44
Abnormal EEG				
Epileptiform spikes	13/25 (52%)	8/15 (53%)	5/10 (40%)	1.00
Exclusive temporal discharges	8/13 (62%)	5/8 (63%)	3/5 (60%)	1.00
Abnormal background/encephalopathy	25/25 (100%)	15/15 (100%)	10/10 (100%)	
MRI				
Bilateral temporal lobe involvement	25 (100%)	15/25 (100%)	10/10 (100%)	
Asymmetry	8/25 (32%)	5/15 (33%)	3/10 (30%)	1.00
Claustrum involvement	9/25 (38%)	6/15 (40%)	3/10 (30%)	0.69
Hippocampal sclerosis +/− atrophy^3^	8/12 (66%)	3/6 (50%)	5/6 (83%)	0.54
Cerebral atrophy	6/12 (50%)	3/6 (50%)	3/6 (50%)	
CSF				
WCC >5/mm^3^	7/24 (29%)	4/15 (27%)	3/9 (33%)	1.00
Raised protein >0.5 mg/mL	4/23 (17%)	1/15 (7%)	3/8 (38%)	0.10
Oligoclonal bands	5/19 (26%)	3/11 (27%)	2/8 (25%)^4^	1.00
Serum antibodies				
Anti‐GAD	3/14 (21%)	0/5	3/9 (33%)^5^	0.26
Anti‐Hu	2/15 (13%)	2/9 (22%)	0/6	0.49
NMDA‐R	2/25 (8%)	2/13 (15%)	0/10	0.49
ANTI‐TPO	4/10 (40%)	0/3	4/7 (57%)^5^	0.20
CSF antibodies				
Anti‐GAD	2/14 (14%)	0/5	2/9 (22%)	0.51
NMDA‐R	1/25 (4%)	1/13 (8%)	0/10	1.00

*p* values calculated using Fisher's Exact Test. NMDA‐R, *N*‐methyl‐d‐aspartate receptor; PICU, pediatric intensive care unit.

^1^ Prodromal symptoms include antecedent infection and symptoms prior to presentation to hospital, and their duration (see supplemental table).

^2^ Reasoning or processing difficulties.

^3^ Twelve cases had repeat imaging >6 month from initial scan (6 cases <12 years and 6 cases 12–18 years).

^4^ No serum paired sample received.

^5^ One patient overlapped anti‐TPO and GAD. In this case neither were deemed pathogenic.

**Table 2:** Treatment and outcome in 25 cases of LE.

	All patients (*N* = 25)	Patients <12 years old (*n* = 15)	Patients 12–18 years old (*n* = 10)	*p* value
Immunomodulation treatment				
Steroids	23/25 (92%)	14/15 (93%)	9/10 (90%)	1.00
IV/PO methylprednisolone	17/25 (68%)	10/15 (66%)	7/10 (70%)	1.00
Prednisolone course	7/25 (28%)	5/15 (33%)	2/10 (20%)	0.66
Prednisolone weaning dose	14/25 (56%)	7/15 (47%)	7/10 (70%)	0.41
Pulsed dexamethasone	5/25 (20%)	5/15 (33%)	0/10	0.06
Time to steroid weeks (range)	1 (1–52)	1 (1–52)	2 (1–20)	0.41
IVIg	14/25 (56%)	7/15 (47%)	7/10 (70%)	0.38
Plasma exchange	7/25 (28%)	3/15 (20%)	4/10 (40%)	0.44
Rituximab	15/25 (60%)	10/15 (67%)	5/10 (50%)	1.00
MMF	4/25 (16%)	2/15 (13%)	2/10 (20%)	1.00
Cyclophosphamide	3/25 (12%)	2/15 (13%)	1/10 (10%)	0.40
Alemtuzumab	1/25 (4%)	0/15	1/10 (10%)	1.00
Natalizumab	1/25 (4%)	1/15 (7%)	0/10	1.00
Tocilizumab	1/25 (4%)	1/15 (7%)	0/10	
Last review months (range)	24 (6–108)	36 (18–90)	24 (6–108)	
Outcome at last review				
Refractory seizures^1^	13/25 (52%)	7/15 (47%)	6/10 (60%)	0.69
Non‐epileptic seizures	4/25 (16%)	1/15 (7%)	3/10 (30%)	0.27
Memory impairment	16/25 (64%)	8/15 (53%)	8/10 (80%)	0.23
Additional Cognitive impairment^2^	11/25 (44%)	6/15 (40%)	5/10 (50%)	0.70
MRS				
Score 0	7/25 (28%)	5/15 (33%)	2/10 (20%)	0.66
Score 1	5/25 (20%)	2/15 (13%)	3/10 (30%)	0.36
Score 2	7/25 (28%)	4/15 (27%)	3/10 (30%)	1.00
Score 3	3/25 (12%)	2/15 (13%)	1/10 (10%)	1.00
Score 4	2/25 (8%)	1/15 (7%)	1/10 (10%)	1.00
Score 5	0/25	0/15	0/10	
Score 6	1/25 (4%)	1/15 (7%)	0/10	1.00
Score ≥3	6/25 (24%)	4/15 (27%)	2/10 (20%)	1.00

*p* values calculated using Fisher's exact test. LE, limbic encephalitis; MMF, mycophenolate mofetil; mRS, modified Rankin Scale; IVIg, intravenous immunoglobulin.

^1^ Refractory as listed in diagnosis list at final follow‐up or seizures despite 2 or more anti‐seizure medication.

^2^ Reasoning or processing speed difficulties.

### Clinical features

Prodromal symptoms were reported in 18/25 (72%) with most common symptoms being fever (*n* = 13), headache (*n* = 10), and vomiting (*n* = 4). Patients under the age of 12 years were more likely to present with fever (11/15 vs. 2/10, *p* = 0.02, Fisher's exact). One patient was found to be SARS‐CoV‐2 positive on respiratory PCR when screened for admission and had positive SARS‐CoV‐2 IgG serology 3 weeks later. All cases presented with seizures, of which 15/25 (60%) required PICU admission for status epilepticus. The next most common presenting features were short‐term memory deficits (*n* = 20; 80%), additional cognitive deficits (*n* = 16; 64%), or behavioral change (*n* = 19; 76%). Visual and auditory hallucinations occurred in 6/25 patients (24%). The median duration of hospital admission was 21 days (range 4–120 days; IQR 11, 30 days) (Tables S1–S4).

### Paraclinical features

**Figure 1:**
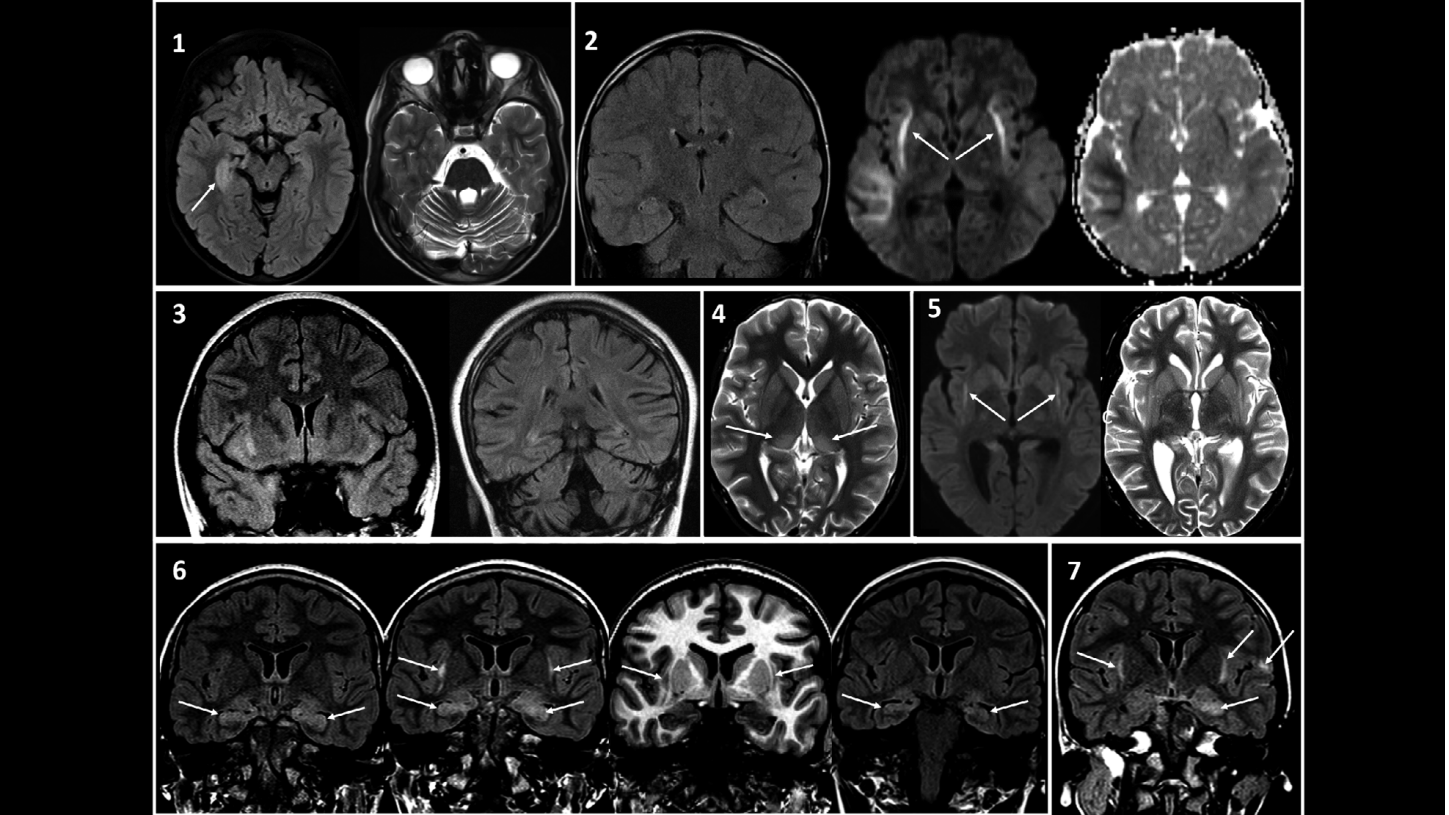
Spectrum of neuroimaging changes in LE patients. 1, Case 11; 11‐year‐old boy presenting with seizures, memory impairment, and behavioral change on a background of sensory axonal neuropathy. Initial FLAIR and axial T2‐weighted MRI sequences demonstrated marked swelling and abnormal hyperintense T2 signal of both hippocampi (asymmetrical R > L). There was moderate cerebellar atrophy. Repeat scan (not shown) at 6 weeks noted slight hyperintensity of the hippocampi with stable cerebellar volume loss. 2, Case 8; 9‐year‐old boy presented with seizures. Coronal T2W, axial diffusion‐weighted imaging (DWI), and ADC (apparent diffusion coefficient) map demonstrates symmetrical signal abnormality and diffusion restriction in the claustra adjacent to the external capsules and the juxtacortical white matter of the right temporal lobe. At 2 years (image not shown), there was progression of diffuse cerebellar and cerebral atrophy. 3, Case 17; 12‐year‐old girl presented with seizures, headache and ataxia. Coronal T2W FLAIR image shows bilateral asymmetric signal abnormalities in the amygdala and hippocampus as well as in the orbital frontal gyrus on the right side. Follow‐up imaging at 2 years identified cerebellar atrophy and bilateral hippocampal sclerosis. 4, Case 7; 8‐year‐old boy presented with seizures and fever. Axial T2WI shows bilateral high signal changes of the posterolateral thalamus (arrows) and claustrum. 5, Case 19; 14‐year‐old boy presented with confusion and seizures. Axial DWI and T2WI shows symmetrical diffusion restriction of the claustrum (arrows) and right posterior perisylvian cortical signal change. At 2 years, there was overall brain atrophy and bilateral mesial temporal sclerosis. 6, Case 23; 15‐year‐old girl presented with seizures, delirium, and abnormal behavior. Initial coronal T2 FLAIR image demonstrates bilateral hippocampal and amygdala signal abnormalities (arrows). Repeat FLAIR and high‐resolution volumetric T1W image performed a week later for ongoing confusion and clinical deterioration demonstrate new claustrum changes bilaterally (arrows). FLAIR imaging a year later showed partial resolution of the signal changes with hippocampal volume loss. 7, Case 10; 10‐year‐old girl presented with status epilepticus. Coronal T2 FLAIR image demonstrates asymmetrical signal changes in the hippocampi and claustrum, with additional patchy cortical changes in the frontal lobes bilaterally (arrow). This figure created for the purposes of this manuscript (with permission to reuse) by the following co‐authors: Kshitij Mankad and Ata Siddiqui. FLAIR, fluid‐attenuated inversion recovery; LE, limbic encephalitis.

MRI abnormalities are summarized in Table 1 and illustrated in Figure 1. As per the inclusion criteria, bilateral mesial temporal changes were seen in all cases, of which 8/25 (32%) were asymmetric, with claustrum involvement in 9/25 patients (38%). EEG demonstrated abnormal background in all cases, with epileptiform discharges reported in 13/25 (52%).

Initial CSF study showed lymphocytic pleocytosis in 7/24 (29%) patients, with median white cell count of 12.5 × 10^6^/L (IQR 7, 15), and elevated CSF protein concentration in 4/23 (17%). Intrathecal oligoclonal bands were identified in 5/19 (26%) patients. All patients had serum anti‐NMDAR Ab and paraneoplastic antibody testing. Testing was performed for anti‐Yo/anti‐Ri (*n* = 15), LGI1 (*n* = 17), CASPR2 (*n* = 17), glycine (*n* = 10), and GABA(B)R‐ab (*n* = 13) antibodies but were negative. Serum antibodies against intracellular antigens (anti‐Hu) were identified in 2/15 children; one of these children had relapsing neuroblastoma and unfortunately died. The second patient had pre‐existing peripheral axonal neuropathy since infancy and whole‐body MRI did not identify an underlying malignancy. He remains under regular tumor surveillance. Two patients (tested *n* = 14) had GAD antibodies in both the serum and CSF; patient 17 had serum GAD antibodies >2000 IU/mL and CSF GAD antibodies >50,000 IU/mL; patient 25 had serum GAD antibodies >2000 IU/mL and CSF GAD antibodies >1,000,000 IU/mL. Two patients with clinical phenotype of anti‐NMDAR encephalitis were positive for anti‐NMDAR Ab in the serum (one also positive in the CSF and second one not tested). Four patients, out of 10 tested, had antibodies to thyroid peroxidase (anti‐TPO), ranging from 178 to 541 IU/mL (normal range 0–150). All were euthyroid at presentation.

### Immune therapy

First‐line immunotherapy was given to 24/25 (96%) patients: steroid treatment in 23/25 (92%), IVIg in 14/25 (56%) and plasma exchange in 7/25 (28%). High‐dose methylprednisolone for 3–5 days (17/25) was the most frequent steroid preparation. Pulsed dexamethasone was used in 5/15 (33%) children under the age of 12 years and was the only therapy used in one case. In 23/25 cases, the median time to first administration of steroids was 1 week (range 1–52 weeks; IQR 1, 2 weeks). The late commencement of steroids was in a child with underlying neuroblastoma. Initial management was neuroblastoma treatment, but symptoms did not resolve, and intrathecal steroids were administered a year after presentation.

Seventeen out of 25 (68%) cases received further immunotherapy, most frequently rituximab 15/25 (60%), which was administered at a median time of 24 weeks from presentation (range 3–184 weeks; IQR 10, 52 weeks). Only one patient received rituximab within 4 weeks from symptom onset. Other therapies given were; mycophenolate mofetil in 4/25 (16%), cyclophosphamide in 3/25 (12%), alemtuzumab in 1/25 (4%), natalizumab in 1/25 (4%), and tocilizumab in 1/25 (4%).

### Disease course and outcome

**Figure 2:**
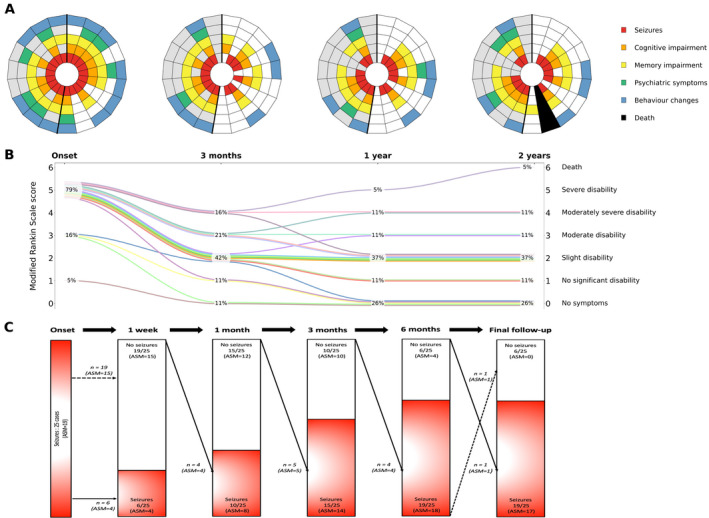
Symptoms and modified Rankin Scale (mRS) scores at presentation, 3 months, 1 year, and 2 years in 19 cases of LE. Seizures and use of ASM at onset, 1 week, 1 month, 3 months, 6 months, and final follow‐up in 25 cases. (A) Each radial segment represents one patient, arranged clockwise from youngest to oldest. Patients <12‐years‐old are shown in the section with white background, and those ≥12‐years‐old in the section with gray background. (B) mRS scores at each timepoint are shown in the middle figure. Each line represents one patient. The proportion with each mRS score at each timepoint is displayed. Six patients had not reached the 2‐year follow‐up. (C) Seizure occurrence and ASM in all 25 patients at each timepoint are shown in the bottom figure. Arrows demonstrate number of cases with change in seizure occurrence and use of ASM over the first 6 months and at final follow‐up (range 6–108 months). One patient that died had seizures requiring PICU admission prior to death. This is reported as seizures at final‐follow‐up. This figure created for the purposes of this manuscript (with permission to reuse) by the following co‐authors: Michael Eyre, Saraswathy Sabanathan, and Ming Lim. LE, limbic encephalitis; PICU, pediatric intensive care unit; ASM, anti‐seizure medication.

**Figure 3:**
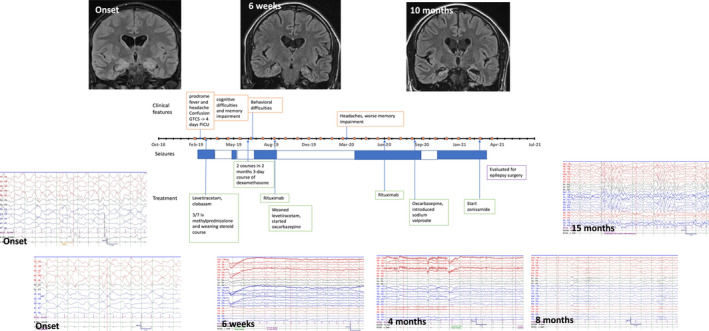
Case vignette and timeline of LE evolution in a 14‐year‐old boy (Case 19). Prodromal symptoms precede seizure onset in a 14‐year‐old boy with LE. Seizure management required PICU admission. Initial coronal T2 FLAIR demonstrated peripheral hyperintensity of the hippocampi and amygdala bilaterally, and interictal EEG had R > L amplitude emphasis at times, with moderate encephalopathy. Prompt treatment with steroids and ASMs resulted in 2 months seizure freedom and improvement in EEG (return of age‐appropriate posterior‐dominant rhythm) at 6 weeks. MRI at 6 weeks demonstrated interval resolution of the previously seen swelling and restricted diffusion of right posterior perisylvian parenchyma and hippocampi, with volume loss and minimal hyperintense signal of hippocampi. There was persistent hyperintense signal of claustrum bilaterally. EEG at 4 months had occasional intermittent slow activity over the R > L posterior region with age‐appropriate posterior dominant rhythm and no encephalopathy. Resurgence of seizures approximately 5 months after presentation was preceded by deterioration in behavior. Treatment with rituximab elicited a good response with further 7 months seizure freedom. At 8 months post‐presentation (and 4 months after 1st rituximab dose), background EEG during sleep was largely symmetrical and continuous within normal limits However, headaches and worsening memory impairment heralded breakthrough of seizure control approximately 13 months since presentation. EEG at 15 months post presentation was abnormal with mild slowing, occasional generalized epileptiform activity, and frequent generalized delta rhythmic activity both maximal over the frontal areas. This was consistent with the clinical picture of worsening cognition and memory impairment. Brain MRI at 10 months demonstrated generalized low brain parenchymal volume in addition to bilateral hippocampal volume loss and altered signal. Despite ASM optimization, there was failure to regain seizure control and epilepsy surgery referral has been initiated. The case highlights the initial “honeymoon” period of seizure freedom followed by return of seizures which appear refractory to ASMs. This figure created for the purposes of this manuscript (with permission to reuse) by the following co‐authors: Omar Abdel‐Mannan, Saraswathy Sabanathan, Krishna Das, and Yael Hacohen. FLAIR, fluid‐attenuated inversion recovery; LE, limbic encephalitis; PICU, pediatric intensive care unit; ASM, anti‐seizure medication.

Regular anti‐seizure drugs (ASMs) were commenced during admission in 19/25 (76%) patients. Immune therapy was administered to 24 patients of which 4/24 (17%) had no further seizures. Seizures continued in 6/25 (24%) with re‐emergence of seizures in 15/25 (60%) at a median time of 12 weeks (range 3–52 weeks; IQR 8, 12 weeks) (Fig. 2). Figure 3 demonstrates the evolution of seizures in a 14‐year‐old boy (Case 19; Table S3); initial seizure acquiescence for 2 months before breakthrough seizures, with further seizure control for 7 months following escalation of immune therapy (rituximab), before emergence of refractory seizures. In 11 cases with 2‐year ASM data, the median number of ASMs used were 3 (range 1–4). At last follow‐up, 13/25 (52%) had refractory seizures (defined as ongoing seizures despite two medications). Univariate logistic regression analysis demonstrated that there was a significant association between refractory seizures at final follow‐up and non‐white ethnicity (*p* = 0.04) and intensive care admission (*p* = 0.004). No significant association was detected between refractory seizures at last follow‐up and age, sex, epileptiform discharges on EEG, abnormal CSF at presentation, rituximab therapy, time from initial presentation to steroid treatment and time from initial presentation to escalation of immune therapy (IVIG/PLEX) (Table S6).

The median length of follow‐up from first clinical presentation was 24 months (range 6–108 months; IQR 18, 48 months). At final follow‐up, memory impairment was reported in 16/25 (64%) and additional cognitive impairment in reasoning or processing was reported in 11/25 (44%); two children moved from mainstream to special schools. Six out of 25 (24%) children had mRS scores of 3 or more, with the main health burden being refractory seizures. In 19 cases with complete 2‐year follow‐up, the greatest reduction in the five main symptoms (seizures, memory impairment, other cognitive impairment, behavior change, psychiatric symptoms) and improvement in mRS scores occurred within the first 3 months (Fig. 2). Beyond this time, difficulties tended to persist despite further immunomodulation. Three patients' mRS scores progressively worsened after the first 3 months; an 11‐year‐old boy (Case 13; Table S2) had progression of cognitive impairment (verbal comprehension and processing speed) by 23 months, an 11‐year‐old girl (Case 14; Table S2) had progression of underlying neuroblastoma associated with refractory seizures by 20 months and a 12‐year‐old girl (Case 17; Table S3) had progressive cognitive deterioration (verbal comprehension and memory impairment) by 53 months. Univariate logistic regression analysis demonstrated that there was a significant association between cognitive impairment at final follow‐up and intensive care admission (*p* = 0.03). No significant predictors were detected for mRS score of 3 or more at final follow‐up (Table S6).

In patients with initial claustrum changes on MRI (*n* = 9) compared to those with no claustrum changes (*n* = 16); there was no difference in memory impairment (*p* = 0.15), cognitive (*p* = 0.2), psychiatric (*p* = 0.35) or behavior symptoms (*p* = 0.63) at presentation, nor in mRS at last review (*p* = 0.97, Mann–Whitney *U*). In those who had follow‐up MRI scans (range 3 weeks to 93 months), sclerosis or atrophy of the hippocampus was identified in 8/12 (66%) (Fig. 1).

Neuropsychological assessments were carried out in 9/24 (38%). Median time to first assessment was 12 months from presentation (range 1–48 months; IQR 5, 18). Five cases had more than one assessment. Below average scores were identified in either memory, reasoning, or processing speed in 8/9 (89%) of cases. Very low scores (<5th percentile) in measures of memory were reported in 6/9 (67%) (Table S5).

A higher proportion of children with refractory seizures (9/14; 64%), memory impairment (10/16; 63%) and additional cognitive impairment (7/11; 64%) received rituximab. In patients who received rituximab (*n* = 15) compared to those who did not (*n* = 10), there was no difference in mRS (*p* = 0.58, Mann–Whitney *U*), memory impairment (*p* = 1.0), cognitive outcomes (*p* = 1.0), or refractory seizures (*p* = 1.0) at last review.

## Discussion

In this retrospective observational study of 25 children with LE, we demonstrate that the diagnosis is associated with significant morbidity and adverse outcomes. Patients typically required intensive care for status epilepticus in the acute illness, followed by improvements in seizure control and functional independence over the first 3 months, then relatively little change thereafter. At 2 years; more than a fifth of patients had an mRS ≥3 (moderate disability), over half of patients had refractory seizures, over half had memory impairment and over 40% had additional cognitive difficulties in reasoning or processing speed. The poor outcomes are in keeping with follow‐up data in LE adult patients, reporting mRS score ≥3 in 35/80 (44%) at last follow‐up (median follow‐up of 22.5 months).^18^ By contrast, in anti‐NMDAR encephalitis children typically have substantial recovery with only 10% having moderate or severe deficits in long‐term follow‐up,^19^ despite the severity of the acute presentation. Refractory seizures were seen more frequently in non‐White patients and may be indicative of a more severe disease course. Similar findings were also reported in other antibody‐mediated syndromes such as aquaporin‐4 antibody‐positive neuromyelitis optica spectrum disorder.^20^

Consistent with the literature in children,^21^ none of the patients in our cohort were positive for LGI1/CASPR2 antibodies, and only 2/25 positive for any neuronal surface antibodies (both NMDAR‐Ab). LGI1 antibody encephalitis‐Abs is extremely rare in children and seldom presents with the phenotype reported in adults.^22^ LE has been reported in association with intracellular antibodies such as anti‐Hu, CRMP5, and Ma2.^23^ Antibodies against these intracellular antigens are not considered directly pathogenic and are more likely the result of immune recognition of tissue destruction.^23^ Detection of these antibodies may suggest the presence of cytotoxic T‐cell‐mediated mechanisms contributing to disease progression. Two out of 15 children in our study had anti‐Hu (Hu‐Abs) antibodies, one of which had relapsing neuroblastoma. In adult patients, Hu‐Abs are the most frequent onconeural antibodies associated with paraneoplastic neurological syndromes,^24^, ^25^ but are exceedingly rare in children.^26^ Only 8 out of 251 (3%) patients with Hu‐Abs in a French retrospective study were under 18 years old^26^; two of these children had neuroblastoma and opsoclonus‐myoclonus, with the other six presenting with non‐paraneoplastic LE. It is possible that our patient with the long‐standing axonal neuropathy and anti‐Hu antibodies, had LE due to an undiagnosed neuroblastoma that spontaneously regressed. An underlying malignancy is seen in 42% of seronegative adult LE patients and associated with poorer prognosis.^6^ In children with immune‐mediated LE, although underlying malignancy is rare, it can be associated with an aggressive disease course refractory to treatment compared to non‐neoplastic antibody mediated LE.^27^, ^28^ Recent updated diagnostic criteria for paraneoplastic neurologic syndromes list LE as a high‐risk phenotype, that is, >70% associated with cancer. As a result, regular tumor surveillance is clinically required.^23^ Two out of 14 children (both >12 years old) in our cohort had serum and CSF anti‐GAD antibodies. One had pre‐existing type 1 diabetes mellitus (DM) and the second developed type 2 DM after presentation with LE, with a 2‐year mRS score of 4. Although rare in children, the management of anti‐GAD‐associated LE is challenging and prognosis is often poor.^29^ Anti‐TPO was detected in four children, but its pathological relevance and clinical utility in LE is controversial.^30^

In this study, PICU admission was required (predominantly for seizure management) in 16/25 (64%) children, higher than up to 48% reported in pediatric anti‐NMDAR encephalitis.^19^, ^31^ However subsequent recovery was faster, with a median duration of hospitalization 21 days (range 4–120, data available in 19 cases) compared to 56 days (range 13–336) in a cohort of 20 children with anti‐NMDAR encephalitis.^19^ In addition, 15 patients had a “honeymoon” period of seizure freedom of at least 1 month, followed by return of seizures which remained refractory to ASMs in 14 patients at last follow‐up. An important consideration when managing these complex patients is that LE is typically a monophasic disease, therefore when seizures and cognition worsen months or years following the acute event, the disease pathobiology may be different. This may be a consequence of ongoing immune‐mediated inflammation, scarring from resolution of inflammation or both. In our cohort, two patients were referred for complex epilepsy management (ketogenic diet and vagal nerve stimulation) after a lack of response to two ASMs.

The involvement of extra‐limbic areas may occur in patients with LE, termed as “LE plus” in the adult literature.^32^ Although this term has not previously been used in pediatric LE literature, in our cohort, 38% of children had changes outside the limbic system on MRI with striking claustrum involvement, and in 6/12 (50%) cerebral atrophy was seen at follow‐up scans. The claustrum is a thin gray matter structure, unique to mammals, embedded in the cerebral white matter between the insular cortex and putamen, with extensive connections to diverse cortical areas.^33^ Its function remains largely unknown, but it is thought to play an important role in attentional control, and so is of potential relevance for the cognitive difficulties observed in this cohort of LE patients.^34^

Cognitive impairment is not always apparent at disease onset in LE, which can result in potential treatment delay^35^; this is of major concern given the possibility of a therapeutic window with early immune therapy potentially preventing long‐term cognitive deficits.^36^ Over half of our cohort had memory impairment and over 40% had additional problems identified in reasoning, or processing speed. This is similar to the reported neuropsychological outcomes of pediatric anti‐NMDAR encephalitis patients, who have persistent cognitive deficits (with observed lower scores in sustained attention and speed) despite excellent functional recovery and quality of life scores.^37^ In adults with anti‐NMDAR encephalitis, the hippocampus appears to play a critical role in mediating the effect of severe disease on memory performance.^38^ MRI changes and particularly hippocampal lesions in anti‐NMDAR encephalitis may be associated with worse outcomes.^39^ In our study, two anti‐NMDAR encephalitis cases, a 6‐year‐old boy (Case 2; Table S1) and an 11‐year‐old girl (Case 15; Table S2), were promptly treated within 1 week of presentation with steroids, and had good MRS scores (score = 0) at last follow‐up. The 11‐year‐old girl relapsed at 45 months and received rituximab. She had persistent difficulties with verbal memory abilities noted on formal neuropsychological testing 4 years after presentation (Table S5).

Early initiation of immune therapy with rituximab (i.e., within 4 weeks) has been shown to result in a more favorable prognosis for children with conditions such as anti‐NMDAR encephalitis; a multicenter study of 144 children with autoimmune and inflammatory CNS disorders demonstrated that in 39 children with anti‐NMDAR encephalitis, 92% had mRS 0–2 at last follow‐up in the “early” treated group (those who received rituximab within 4 weeks of symptoms onset) compared to only 57% in the “late” treated group.^40^ Improvement in mRS score and favorable mRS scores were associated with additional monthly rituximab therapy and partial response to first line immunotherapies. In this current cohort, a higher proportion of patients with persistent symptoms received rituximab after 4 weeks (14/15; 93%) which may reflect a treatment paradox where more severe cases receive escalation of therapies. Hence, we were unable to evaluate the impact of “early” versus “late” rituximab in LE. Of note, the one patient (antibody‐negative) who received rituximab within 4 weeks in our cohort has made complete recovery 2 years from onset and has no evidence of brain atrophy on serial MRI imaging. The greatest improvement in symptoms in this current cohort occurred within 3 months of presentation; and 3 months was also the median time for seizure recurrence. Therefore, the first 3 months may be a critical window for immune modulation in LE.

Our study is limited by its retrospective design, with a small sample of patients given the rarity of the condition and a lack of standardized data collection as a “real‐world” observational cohort. Furthermore, despite the distinct clinical phenotype, LE is likely to represent many immuno‐pathobiological entities. In the current era of precision‐targeted therapies, future humoral and cellular biomarker identification are required to gain insight into the pathogenesis for early diagnosis and optimal treatment of LE.

## Conflict of Interest

Cheryl Hemingway has received educational and travel grants from Merck Serono and Bayer and Biogen.

Ming Lim receives research grants from Action Medical Research, the DES society, GOSH charity, National Institute for Health Research, MS Society, and SPARKS charity; receives research support grants from the London Clinical Research Network and Evelina Appeal; has received consultation fees from CSL Behring, Novartis and Octapharma; received travel grants from Merck Serono.

## Author Contributions

Saraswathy Sabanathan and Omar Abdel‐Mannan: Drafting/revision of the manuscript for content, including medical writing for content; major role in the acquisition of data; study concept or design; analysis or interpretation of data. Kshitij Mankad, Ata Siddiqui, and Krishna Das: Drafting/revision of the manuscript for content, including medical writing for content; major role in the acquisition of data. Lucinda Carr, Christin Eltze, Jon Gadian, Cheryl Hemingway, Marios Kaliakatsos, Rachel Kneen, Deepa Krishnakumar, Bryan Lynch, Amitav Parida, Thomas Rossor, Micheal Taylor, and Evangeline Wassmer: Drafting/revision of the manuscript for content; major role in the acquisition of data. Michael Eyre: Drafting/revision of the manuscript for content, analysis, or interpretation of data, and drafting/revision of the manuscript for content, including medical writing for content. Sukhvir Wright: Drafting/revision of the manuscript for content, including medical writing for content; major role in the acquisition of data. Ming Lim and Yael Hacohen: Drafting/revision of the manuscript for content, including medical writing for content; major role in the acquisition of data; study concept or design; and analysis or interpretation of data.

## Supporting information


**Tables S1–S4.** Clinical history, demographics, treatment, and follow‐up information for 25 cases of limbic encephalitis.

**Tables S5.** Cognitive profile of nine cases of limbic encephalitis who had standardized tests administered.

**Tables S6.** Univariate analysis of risk factors for refractory seizures, cognitive impairment, and mRS score of 3 or more in 25 cases of limbic encephalitis.

## Data Availability

Data are available upon reasonable request. The deidentified participant data are available from the corresponding author y.hacohen@ucl.ac.uk. Both centre and department have to give the permission to reuse the database.
