# Supplementary material for: Clinical features, investigations, and outcomes of pediatric limbic encephalitis: A multicenter study
Source: Ann Clin Transl Neurol. 2022 Jan 11;9(1):67–78. doi: 10.1002/acn3.51494 (PMC8791799; doi:10.1002/acn3.51494)
Supplement: Supplementary file 1 — Tables S1–S4. Clinical history, demographics, treatment, and follow‐up information for 25 cases of limbic encephalitis. [file ACN3-9-67-s001.docx]

Supplementary data (table e-1)

|  | Case 1 | Case 2 | Case 3 | Case 4 | Case 5 | Case 6 | Case 7 | Case 8 |
| --- | --- | --- | --- | --- | --- | --- | --- | --- |
| Age at disease onset | 4 | 6 | 6 | 7 | 7 | 7 | 8 | 9 |
| Sex | Male | Male | Female | Male | Female | Female | Male | Male |
| Ethnicity | White British | White | Pakistani | Not recorded | Asian | Other Ethnic Group | Other Ethnic group | Bangladeshi |
| Pre-existing medical conditions | Speech delay | None | None | None | None | None | None | None |
| Antecedent history | Covid+ve PCR (asymptomatic) | Sore throat and headache | Fever 2 weeks prior | Cough | None | Coryzal 4 days, GP treated for tonsillitis with Pen V | PUO 11 days 4 weeks prior to admission | Fever and diarrhoea 4 days prior |
| Presenting symptoms | Fever and GTCS | Acute confusional state, hyperactive, aphasia, poor concentration | Confusion, visual hallucination, encephalopathy | Headache, behaviour change, auditory hallucinations, cognitive concerns, encephalopathy, memory impairment, expressive language difficulty, GTCS and focal seizures | Vomiting, fever, non-convulsive status | Focal seizures and GTCS. Expressive dysphasia, hallucination and disorientation | GTCS with fever | GTCS, deteriorating verbal skills, fluctuating GCS |
| Initial treatment (time from first presentation) | Dexamethasone 15mg/m2 dose 3 days monthly for 3 months (2 weeks) | IVIg for a total of 5 days (1 week), pulsed methylprednisolone (30mg/kg) for 3 days (2 week), followed by oral prednisolone for 4 weeks | IV methylprednisolone for 5 days (1 week), weaning prednisolone, IVIg (2 weeks) | IV methylprednisolone for 5 days, weaning prednisolone (1 week), IVIg (1 week), plasma exchange (1 week) | None | IV methylprednisolone 5 days and oral course (1 week), plasma exchange (2 week), IVIg (3rd week) dexamethasone for 3 days every month (from 1st month) for 3 months | IV methylprednisolone 5 days and weaning prednisolone (1 week) | IVIg and IV methylprednisolone 5 days and weaning prednisolone (1 week) |
| Further treatment (time from first presentation) | Rituximab (2 months) | None | None | Rituximab and cyclophosphamide (1 month) | None | MMF (6-12 months), rituximab (1 year), Tocilizumab (18 months) | Rituximab (2 month) | Rituximab (1 year), MMF (1 year) |
| Initial seizure freedom | None | No further seizures | None | None | None | 4 months | 11 months | 2 months |
| AED on discharge | None | None | Carbamazepine, levetiracetam, clobazam | Levetiracetam | Topiramate, phenytoin, clobazam | Levetiracetam, phenobarbitone, sodium valproate | Levetiracetam | Levetiracetam, phenytoin |
| Time to first AED  (1^st^ AED) | Not applicable | None | Admission | Admission | Admission | Admission | Admission | Admission |
| Time to 2nd AED (2^nd^ AED) | Not applicable | Not applicable | Admission | NA | Admission | Admission | 1 year (sodium valproate) | 2 months (oxcarbazepine) |
| AED at 2 years (number) | Not yet 2 years | None | Brivaracetam, clobazam, zonisamide, oxcarbazepine (4) | Clobazam (1) | Topiramate, lacosamide, clobazam, VNS (4) | Phenobarbitone, sodium valproate, clobazam (3) | Not yet 2 years | Sodium valproate, oxcarbazepine, perampanel (3) |
| Time to last review | 6 months | 108 months | 24 months | 36 months | 84 months | 24 months | 15 months | 54 months |
| Problems at last review | Limbic encephalitis, Previous speech delay | None | Intractable focal seizures | Refractory seizures, Evolving neurocognitive sequelae with abnormal language  Non-specific inflammatory bowel disorder diagnosed histologically | Autoimmune encephalitis, learning difficulties, drug resistant seizures | Antibody negative autoimmune encephalitis, seizure disorder, behaviour difficulties, NEAD | Autoimmune encephalitis | Intractable seizures PEG fed Acquired learning difficulties Recurrent hyponatraemia Drooling |
| Neurology Medication at last review | None | None | Brivaracetam, clobazam, zonisamide, oxcarbazepine | Clobazam | Levetiracetam, oxcarbazepine, topiramate | Phenobarbitone, sodium valproate, clobazam, aripiprazole, promethazine, clonidine | Sodium valproate, levetiracetam | Alimemazine,  clobazam,  Melatonin, oxcarbazepine, perampanel, sodium valproate |
| mRS at last review | 0 | 0 | 2 | 2 | 2 | 2 | 0 | 4 |

ADL – activities of daily living, GCS – Glasgow coma score, GTCS – generalised tonic clonic seizures, IV – intravenous, IVIg – immunoglobulins, KD – ketogenic diet, MMF – Mycophenolate mofetil, mRS – modified Rankin Score, NA – not available, NEAD – non-epileptic attack disorder, PEG - percutaneous endoscopic gastrostomy, PUO – pyrexia of unknown origin, SLE – systemic lupus erythematosus, VNS – vagal nerve stimulation.

Supplementary data (table e-2)

|  | Case 9 | Case 10 | Case 11 | Case 12 | Case 13 | Case 14 | Case 15 |
| --- | --- | --- | --- | --- | --- | --- | --- |
| Age at disease onset | 9 | 10 | 11 | 11 | 11 | 11 | 11 |
| Sex | Female | Female | Male | Male | Male | Female | Female |
| Ethnicity | Black Other | Mixed | Other White | Black | Black African | Asian | White British |
| Pre-existing medical conditions | Under investigation for neutropenia | None | Sensory motor axonal neuropathy | None | None | Abdominal Neuroblastoma 3 years prior - treated | None |
| Antecedent history | 3 days unpleasant smell, headache, confusion episodes then clusters | 5 day lethargy, headaches knee pain, | None | Flu like illness 2 weeks prior - parainfluenza positive | None | Peripheral neuropathy, weakness, ataxia and movement disorder | None |
| Presenting symptoms | Focal with generalisation seizures | Behaviour change, hallucinations followed by absence seizures that progressed to GTCS | Episodes over 4 months of appearing scared and vacant, with oro-masticatory movements, deterioration in school work, rapid deterioration in motor abilities, short term memory impairment, behaviour change | Aggressive behaviour change, not sleeping. GTCS | Headache, prolonged febrile seizure, memory impairment, dysarthric | 3 months later seizures | 3 week history acute confusion, hallucination, unusual behaviour. 2 weeks later 2 seizures |
| Initial treatment (time from first presentation) | 5 days high dose oral methylprednisolone, 10 days high dose prednisolone (9 months), dexamethasone 3 days monthly pulse for 3 months (started 10th month) | 3 day high dose methylprednisolone (1 week), plasma exchange (2 weeks), 3 days dexamethasone monthly (started 2nd month for 6 months) | 3 days IV methylprednisolone, weaning prednisolone (2 month) | IVIg (1 week), prednisolone (2 weeks) | 5 days high dose IV methylprednisolone, oral prednisolone for 6 weeks then weaned over 6 weeks (1 week)  IVIg (2 weeks) | Intrathecal steroids (1 year) following  treatment for recurrence neuroblastoma | IVIg and prednisolone (1 week), dexamethasone 3 days every month for 3 months (started 2 weeks) |
| Further treatment (time from first presentation) | Rituximab (9 months) | Rituximab (5 months) | Rituximab (3 month) | None | None | Rituximab (12 months), nataluzimab (12 months) cyclophosphamide (12 months) | Rituximab (46 months) at relapse |
| Initial seizure freedom | 2 months | 4 months | 5 months | No further seizures | 3 seizures in first 6 months | 12 months | No further seizures |
| AED on discharge | Levetiracetam | Levetiracetam | None | Levetiracetam | Levetiracetam, phenytoin | Levetiracetam | Levetiracetam |
| Time to first AED  (1^st^ AED) | Admission | Admission | 6 months (sodium valproate) | Admission | Admission | Admission | Admission |
| Time to 2nd AED (2^nd^ AED) | Not applicable | Not applicable | Not applicable | Not applicable | 9 months (topiramate) | Not applicable | Not applicable |
| AED at 2 years (number) | Not yet 2 years | Not yet 2 years | Not yet 2 years | None | Topiramate, levetiracetam (2) | Not alive | None |
| Time to last review | 13 months | 17 months | 10 months | 36 months | 72 months | 18 months | 48 months |
| Problems at last review | Focal seizures ongoing  When frequent focal seizures, memory impaired | Autoimmune encephalitis | Limbic encephalitis, Axonal neuropathy, Cerebellar atrophy | None - discharged | Limbic encephalitis, progressive cerebellar and cerebral atrophy for 2 years post presentation. Progressive cognitive impairment for 2 years. Central hypothyroidism. | PICU admission for status epilepticus. Plan for palliation | NMDAR encephalitis |
| Neurology Medication at last review | Levetiracetam | Levetiracetam, aripiprazole | Sodium Valproate | None | Levetiracetam, lacosamide, clobazam, mycophenolate, | KD, levetiracetam, gabapentin | None |
| mRS at last review | 1 | 1 | 3 | 0 | 3 | 6 | 0 |

Supplementary data (table e-3)

|  | Case 16 | Case 17 | Case 18 | Case 19 | Case 20 | Case 21 |
| --- | --- | --- | --- | --- | --- | --- |
| Age at disease onset | 12 | 12 | 13 | 14 | 14 | 14 |
| Sex | Male | Female | Female | Male | Male | Female |
| Ethnicity | White | Indian | Mixed: Asian/Caucasian | Other Black | White | White British |
| Pre-existing medical conditions | None | None | None | None | None | None |
| Antecedent history | 2 episodes of confusion 6 weeks prior, lethargy, and heavy sensation in the right arm lasting 18 hours. | None | 1^st^ seizure 3 months previously, followed by headaches and change in personality. | Headache, red eye and fever 2 weeks prior to presentation | 2-week history weakness, headaches, and dizziness | URTI in previous week |
| Presenting symptoms | Severe headache, aggressive behaviour, short-term memory loss, and seizures | GTCS, visual and auditory hallucinations, ataxia, headache | Seizures, withdrawn, memory problems | Confusion, GTCS, short term memory difficulties. | Encephalopathy and GTCS, short-term memory impairment, withdrawn, cognitive difficulties | Focal seizures with secondary generalisation and GTCS. Withdrawn, short term memory difficulties, auditory hallucinations |
| Initial treatment (time from first presentation) | 5 days high dose methylprednisolone, IVIg (2 weeks), 4 courses plasma exchange while on high dose oral steroids (2 month - weaned steroids over 4 months) | Oral prednisolone high dose 4 weeks, wean over 6 weeks (started 5 months)  IVIg (6 months)  plasma exchange (12 months)  Serum GAD antibodies 37506 U/ml (<5 U/ml). CSF GAD antibody detected. | 5 days high dose methylprednisolone (1 week), IVIg, (2 weeks) plasma exchange (1 month) | 3 days high dose methyl prednisolone, weaning steroids  (2 weeks) | 3 days IV methylprednisolone high dose (1 week), IVIg (2 weeks) | 5 days high dose IV methylprednisolone (1 week), followed by oral prednisolone for 6 weeks weaned over 6 weeks |
| Further treatment (time from first presentation) | Rituximab (6 months) | Alemtuzumab (6 months)  rituximab (1 year)  MMF (13 months) | MMF (started 2 months) | Rituximab (6 months) | Cyclophosphamide (4 weeks) | Rituximab (3 weeks) |
| Initial seizure freedom | 5 months | None | None | 2 months | 3 months | 2 months |
| AED on discharge | Sodium valproate | none | Levetiracetam | Levetiracetam and weaning plan clobazam | None | levetiracetam |
| Time to first AED  (1^st^ AED) | Admission | 6 weeks (Levetiracetam) | Admission | Admission | Not applicable | Admission |
| Time to 2nd AED (2^nd^ AED) | 6 months - phenytoin | 11 months (Topiramate) | Admission (Phenobarbitone) | 7 months (Oxcarbazepine) | Not applicable | Not applicable |
| AED at 2 years (number) | NA | Sodium valproate, topiramate, phenytoin (3) | Levetiracetam, phenobarbitone (2) | Sodium valproate, zonisamide (2) | NA | None |
| Time to last review | 24 months | 90 months | 36 months | 24 months | 24 months | 18 months |
| Problems at last review | Seizures | Pharmacoresistant focal epilepsy, Cognitive impairment secondary - needs help with ADLs, NEAD, Osteopenia, Insulin dependent diabetes, Hyperlipidaemia. | Intractable focal seizures, low mood, fatigue, anxiety, syncopal events, NEAD | Limbic encephalitis  Pharmacoresistant focal epilepsy | Seizures, short-term memory difficulties  Hypothyroidism | Limbic encephalitis - resolved |
| Neurology Medication at last review | NA | Topiramate  Lacosamide  Clobazam  Azathioprine | Perampanel, clobazam, phenobarbitone, Levetiracetam, MMF | Sodium valproate | NA | None |
| mRS at last review | 1 | 4 | 2 | 2 | 1 | 0 |

Supplementary data (table e-4)

|  | Case 22 | Case 23 | Case 24 | Case 25 |
| --- | --- | --- | --- | --- |
| Age at disease onset | 14 | 15 | 15 | 15 |
| Sex | Female | Female | Female | Female |
| Ethnicity | Indian | White British | White | Black |
| Pre-existing medical conditions | None | Behavioural difficulties, school exclusion | None | Type 1 Diabetes Mellitis  Hypothyroidism |
| Antecedent history | 5 days prior fever, headache | 1 week prodrome of feeling unwell and rundown, known illicit drug use | 1^st^ GTCS 3 months previously | Temporal lobe seizures (sense of deja-vu and odd smell) and headache 4 months prior to hospital presentation |
| Presenting symptoms | Seizures, neuropsychiatric features | GTCS, delirium, hallucinations | GTCS seizure | GTCS seizure |
| Initial treatment (time from first presentation) | 5 days high dose IV methylprednisolone (1 week), followed by oral prednisolone weaned over 6 weeks, plasma exchange and IVIg (1 month) | High dose prednisolone and weaned (1 week), IVIg (3 courses - 3 weeks, 7 months, 8 months) | 3 days methylprednisolone (2 months), high dose prednisolone with weaning course. | IVIg (8 months & 11 months) |
| Further treatment (time from first presentation) | Rituximab (14 months) | None | None | None |
| Initial seizure freedom | 1 month | 3 weeks | No further seizures | 2 weeks |
| AED on discharge | Phenytoin | none | carbamazepine | Levetiracetam |
| Time to first AED  (1^st^ AED) | Admission | 3 weeks (Carbamazepine) | Admission | Admission |
| Time to 2nd AED (2^nd^ AED) | NA | 6 weeks (Topiramate) | Not applicable | Not applicable |
| AED at 2 years (number) | Oxcarbazepine, levetiracetam, clobazam (3) | Carbamazepine, levetiracetam, clobazam, phenytoin (4) | None | Not yet |
| Time to last review | 36 months | 72 months | 24 months | 6 months |
| Problems at last review | Drug resistant epilepsy with temporal semiology Memory, cognitive and difficulties Mood disturbances NEAD  VNS, KD | Poorly controlled SLE, Epilepsy, psychiatric problems | None | Temporal lobe epilepsy secondary to autoimmune limbic encephalitis (suspected anti-GAD encephalitis) |
| Neurology Medication at last review | Levetiracetam,  Oxcarbazepine | Levetiracetam | None | Levetiracetam |
| mRS at last review | 2 | 3 | 0 | 1 |

Supplementary data (table e-5)

|  | Case 2 | Case 6 | | Case 13 | | | Case 15 | | Case 17 | | Case 18 | Case 22 | | Case 23 | Case 24 |
| --- | --- | --- | --- | --- | --- | --- | --- | --- | --- | --- | --- | --- | --- | --- | --- |
| Age at disease onset | 6 | 7 | | 11 | | | 11 | | 12 | | 13 | 14 | | 15 | 15 |
| Sex | Male | Female | | Male | | | Female | | Female | | Female | Female | | Female | Female |
| Ethnicity | Irish | Other Ethnic Group | | Black African | | | White British | | Indian | | Mixed: Asian/White | Indian | | White British | White |
| Assessed from presentation (months) | 24 | 8 | 16 | 1 | 23 | 96 | 5 | 48 | 18 | 53 | 14 | 12 | 36 | 48 | 1 |
| Test used | WISC-IV | BAS-3 | WISC-V & CHAMP | WISC-IV | | WAIS-IV UK | WISC-V | WISC-V & CHAMP | WISC-IV & CMS | WISC-IV & CMS | WISC-V | WISC-V | WISC-V  & CHAMP | Cognitive Linguistic Quick Test | WISC-IV |
| Full scale/ Reasoning | Verbal reasoning average range.  Perceptual Reasoning Borderline impaired range. | Low Average range | Average to low average range. | VCI=27^th^ %ile  Average range | VCI=4^th^ %ile  Low range | VCI=32^nd^ %ile  Average range | Full scale IQ high Average range | Full scale IQ Average range | VCI=19^th^ %ile Average range | VCI =<1^st^ %ile.  Age equivalent 9-11 years | FSIQ =2^nd^ %ile  Very low range. VCI=10^th^ %ile low average range | Average to high average range | FSIQ=66^th^ %ile  Average range | Executive function, language, visuo-spacial, low range. | NA |
| Memory | WMI average. | Very Low range* | Very Low range* | WMI=4^th^  %ile  Very Low range* | WMI=<1^st^ %ile  Very low range* | WMI=9^th^ %ile  Low average range | High average range | Borderline impairmentverbal memory | CMS Average range in all areas | Visual Immediate, Verbal Immediate and Verbal Delayed - Very low range* | WMI=2^nd^ %ile  Very low range* | Low to very low range* | Very low range*  *Anterograde*  *amnesia* | Moderate difficulties | VII score=12^th^ %ile  Low average range  VDI score= 3^rd^ %ile Very low range* |
| Processing | High range. | Very high range | Not completed | PSI=16^th^ %ile  Low average | PSI=<1^st^ %ile  Very low range* | PSI=1^st^ %ile  Very low range* | Average range | Average range | Average range | Not completed | 0.4^th^%ile  Very low range* | Not reported | 37^th^ %ile Average range | Mild difficulties | NA |
| mRS at last review | 0 | 2 | | 3 | | | 0 | | 4 | | 2 | 2 | | 3 | 0 |

%ile – percentile, BRIEF 2 - Behavior Rating Inventory of Executive Function, CHAMP - Child and Adolescent memory profile. CMS - Children’s Memory Scale, FSIQ- Full-Scale IQ, NA – not available, PSI – processing speed index, VCI verbal comprehension index. WAIS-IVUK - Wechsler Adult Intelligence Scale 4th UK Edition. WISC-IV – Wechsler Intelligence Scale for Children 4^th^ edition. WMI: Working memory index, VDI - Verbal delayed Index , VI - Verbal Immediate Index.

*Very low range <5^th^ percentile on

Supplementary data (table e-6)

| Risk factor | Outcome | | | | | |
| --- | --- | --- | --- | --- | --- | --- |
|  | **Refractory Seizures** | | **Cognitive impairment** | | **mRS score of 3 or more** | |
|  | ***Odds ratio (95% CI)*** | ***P value*** | ***Odds ratio (95% CI)*** | ***P value*** | ***Odds ratio (95% CI)*** | ***P value*** |
| Age at disease onset | 0.95 (0.75,1.23) | 0.71 | 1.08 (0.84, 1.40) | 0.63 | 1.13 (0.83, 1.53) | 0.45 |
| Sex | 1.11 (0.23,5.45) | 0.90 | 0.46 (0.09, 2.32) | 0.35 | 0.73 (0.12, 4.59) | 0.74 |
| Ethnicity | 6.42 (1.09,37.7) | **0.04** | 1.31 (0.26, 6.64) | 0.74 | 1.45 (0.21, 9.98) | 0.7 |
| Intensive care admission | 34.7 (3.06, 393.2) | **0.004** | 13.3 (1.32, 134.6) | **0.03** | 3.64 (0.35, 37.5) | 0.28 |
| Epileptiform discharges on EEG | 0.83 (0.17,4.06) | 0.82 | 1.2 (0.25, 5.84) | 0.82 | 0.9 (0.14, 5.65) | 0.91 |
| Abnormal CSF at presentation | 1.31 (0.26,6.64) | 0.74 | 2.5 (0.30, 7.53) | 0.62 | 0.69 (0.1, 4.72) | 0.7 |
| Rituximab therapy | 1.5 (0.30, 7.53) | 0.62 | 1.31 (0.26, 6.64) | 0.74 | 1.45 (0.21, 9.98) | 0.7 |
| Time from initial presentation to steroid treatment | 0.99 (0.98, 1.01) | 0.4 | 0.10 (0.98, 1.01) | 0.79 | 1.0 (0.99, 1.02) | 0.59 |
| Time from initial presentation to escalation of immune therapy (IVIG/PLEX) | 0.10 (0.98, 1.01) | 0.65 | 0.10 (0.99, 1.01) | 0.98 | 1.0 (0.99, 1.02) | 0.6 |
